# International Editor's Update

**DOI:** 10.3201/eid0606.000601

**Published:** 2000

**Authors:** Takeshi Kurata

**Affiliations:** National Institute of Infectious Diseases and Ministry of Health and Welfare, Tokyo, Japan

**Keywords:** Japan, international travel, antibiotics, nosocomial infections,


International Editors update

### International Editor's Update—Japan

**Takeshi Kurata**
National Institute of Infectious Diseases
Ministry of Health and Welfare, Tokyo, Japan

Dr. Kurata, an international editor of this journal, is deputy director of the National Institute of Infectious Diseases and director of the Department of Pathology, University of Tokyo. His research interests focus on viral pathology.

For 20 years after the end of World War II, infectious diseases were endemic throughout Japan, which served during this first postwar phase almost as a museum of communicable diseases. Improvements in socioeconomic conditions, infrastructure (especially water and sewerage systems), and nutrition brought about a rapid reduction in rates of acute enteric bacterial and parasitic infections. The development and clinical application of antibiotics also contributed to this decrease.

During the second postwar period (1965-1985), further advancement in the use of antibiotics led to control of acute enteric bacterial diseases. However, medical advances such as cancer chemotherapy and organ transplantation, along with an increasing elderly population, created a large immunocompromised population and widespread opportunistic infections. The development of new antibiotics was followed by the emergence of pathogens resistant to drugs.

Since 1975, chemicals used in agriculture have been reevaluated to exclude toxic substances; however, decreased use of chemicals in agriculture has led to the reappearance or emergence of ticks and the rickettsia they transmit.

In the third postwar period (1985-present), increased international travel has led to an increase in imported infectious diseases. Travelers returning from other Asian countries and other continents have become ill with foodborne and insect-borne infections, including shigellosis, cholera, and typhoid fever; several thousand cases are reported each year. In addition, contaminated imported foods have been responsible for sporadic illnesses or small outbreaks.

Misuse or overuse of antibiotics has led to the emergence of methicillin-resistant *Staphylococcus aureus*, penicillin-resistant *Streptococcus pneumoniae*, fluoroquinolone-resistant *Pseudomonas aeruginosa*, and vancomycin-resistant enterococci. All hospitals in Japan must now be alert to nosocomial infections caused by these drug-resistant pathogens.

The most important public health problems in modern Japan are massive outbreaks of acute enteric bacterial diseases. These outbreaks are caused by foods prepared commercially on a large scale for school lunches and chain stores. Contamination in a single aspect of preparation has resulted in large single-source foodborne outbreaks. More than 20,000 cases of infections caused by vibrios, Staphylococcus, pathogenic *Escherichia coli*, and Campylobacter have been reported in the past 5 years.

Concerning viral diseases, immunization programs against measles, rubella, and mumps have been mounted, in addition to the successful campaign against polio in the mid-1970s. However, except for polio, the coverage rate for individual vaccines is lower than rates in the United States and Europe, and vaccine-preventable viral illnesses remain at unsatisfactory levels. Viral diarrheal enteritis transmitted through foods such as oysters has also been increasing.

Trends in infectious diseases have changed rapidly in Japan during the past 50 years. Three reports are included in this issue that update the status of tuberculosis, flavivirus infection, and antibiotic resistance in Japan.

Address for correspondence: Takeshi Kurata, National Institute of Infectious Diseases, Toyama 1-23-1, Shinjuku, Tokyo, 162-8640 Japan; fax: 81-3-5285; e-mail: tkurata@nih.go.jp.

**Figure:**
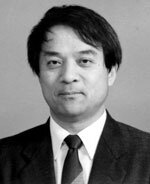
Dr. Kurata, an international editor of this journal, is deputy director of the National Institute of Infectious Diseases and former director of the Department of Pathology, University of Tokyo. His research interests focus on viral pathology.

For 20 years after the end of World War II, infectious diseases were endemic throughout Japan, which served during this first postwar phase almost as a museum of communicable diseases. Improvements in socioeconomic conditions, infrastructure (especially water and sewerage systems), and nutrition, brought about a rapid reduction in rates of acute enteric bacterial and parasitic infections. The development and clinical application of antibiotics also contributed to this decrease.

During the second postwar period (1965-1985), further advancement in the use of antibiotics led to control of acute enteric bacterial diseases. However, medical advances such as cancer chemotherapy and organ transplantation, along with an increasing elderly population, created a large immunocompromised population and widespread opportunistic infections. The development of new antibiotics was followed by the emergence of pathogens resistant to drugs.

Since 1975, chemicals used in agriculture have been reevaluated to exclude toxic substances; however, decreased use of chemicals in agriculture has led to the reappearance or emergence of ticks and the rickettsia they transmit.

In the third postwar period (1985-present), increased international travel has led to an increase in imported infectious diseases. Travelers returning from other Asian countries and other continents have become ill with foodborne and insect-borne infections, including shigellosis, cholera, and typhoid fever; several thousand cases are reported each year. In addition, contaminated imported foods have been responsible for sporadic illnesses or small outbreaks.

Misuse or overuse of antibiotics has led to the emergence of methicillin-resistant Staphylococcus aureus, penicillin-resistant Streptococcus pneumoniae, fluoroquinolone-resistant Pseudomonas aeruginosa, and vancomycin-resistant enterococci. All hospitals in Japan must now be alert to nosocomial infections caused by these drug-resistant pathogens.

The most important public health problems in modern Japan are massive outbreaks of acute enteric bacterial diseases. These outbreaks are caused by foods prepared commercially on a large scale for school lunches and chain stores. Contamination in a single aspect of preparation has resulted in large single-source foodborne outbreaks. More than 20,000 cases of infections caused by vibrios, Staphylococcus, pathogenic Escherichia coli, and Campylobacter have been reported in the past 5 years.

Concerning viral diseases, immunization programs against measles, rubella, and mumps have been mounted, in addition to the successful campaign against polio in the mid-1970s. However, except for polio, the coverage rate for individual vaccines is lower than rates in the United States and Europe, and vaccine-preventable viral illnesses remain at unsatisfactory levels. Viral diarrheal enteritis transmitted through foods such as oysters has also been increasing.

Trends in infectious diseases have changed rapidly in Japan during the past 50 years. Three reports are included in this issue that update the status of tuberculosis, flavivirus infection, and antibiotic resistance in Japan.

